# Patterns of atrophy in pathologically confirmed dementias: a voxelwise analysis

**DOI:** 10.1136/jnnp-2016-314978

**Published:** 2017-05-04

**Authors:** Lorna Harper, Femke Bouwman, Emma J Burton, Frederik Barkhof, Philip Scheltens, John T O’Brien, Nick C Fox, Gerard R Ridgway, Jonathan M Schott

**Affiliations:** 1 Dementia Research Centre, University College London Institute of Neurology, London, UK; 2 Alzheimer Centre, VU University Medical Centre, Amsterdam, The Netherlands; 3 Institute for Ageing and Health, Newcastle University, Newcastle upon Tyne, UK; 4 Department of Radiology and Nuclear Medicine, VU Medical Center, MS Center, Amsterdam, The Netherlands; 5 Department of Brain Repair and Rehabilitation, University College London Institute of Neurology, London, UK; 6 Department of Medical Physics & Biomedical Engineering, University College London Faculty of Engineering Sciences, London, UK; 7 Department of Psychiatry, University of Cambridge, Cambridge, UK; 8 FMRIB Centre, Nuffield Department of Clinical Neurosciences, University of Oxford, Oxford, UK; 9 Wellcome Trust Centre for Neuroimaging, UCL Institute of Neurology, London, UK

**Keywords:** dementia, Alzheimer’s disease, brain atrophy, neuropathology, MRI

## Abstract

**Objective:**

Imaging is recommended to support the clinical diagnoses of dementias, yet imaging research studies rarely have pathological confirmation of disease. This study aims to characterise patterns of brain volume loss in six primary pathologies compared with controls and to each other.

**Methods:**

One hundred and eighty-six patients with a clinical diagnosis of dementia and histopathological confirmation of underlying pathology, and 73 healthy controls were included in this study. Voxel-based morphometry, based on ante-mortem T1-weighted MRI, was used to identify cross-sectional group differences in brain volume.

**Results:**

Early-onset and late-onset Alzheimer’s disease exhibited different patterns of grey matter volume loss, with more extensive temporoparietal involvement in the early-onset group, and more focal medial temporal lobe loss in the late-onset group. The Presenilin-1 group had similar parietal involvement to the early-onset group with localised volume loss in the thalamus, medial temporal lobe and temporal neocortex. Lewy body pathology was associated with less extensive volume loss than the other pathologies, although precentral/postcentral gyri volume was reduced in comparison with other pathological groups. Tau and TDP43A pathologies demonstrated similar patterns of frontotemporal volume loss, although less extensive on the right in the 4-repeat-tau group, with greater parietal involvement in the TDP43A group. The TDP43C group demonstrated greater left anterior-temporal involvement.

**Conclusions:**

Pathologically distinct dementias exhibit characteristic patterns of regional volume loss compared with controls and other dementias. Voxelwise differences identified in these cohorts highlight imaging signatures that may aid in the differentiation of dementia subtypes during life. The results of this study are available for further examination via NeuroVault (http://neurovault.org/collections/ADHMHOPN/).

## Introduction

Cerebral atrophy is a downstream effect of neurodegeneration,^1^ with detectable changes in early^2^ and even presymptomatic^3^ disease stages. Patterns of cross-sectional atrophy are included as core or supporting features in many consensus diagnostic guidelines,^4–8^ and longitudinal atrophy rates are used as outcome measures in clinical trials.^9^ While there is substantial evidence to support the use of patterns of brain volume loss to aid in the differential diagnosis of dementia,^10 11^ much of the evidence base is derived from patients with clinical diagnoses, which may be inaccurate in up to 30% of cases, even in specialist centres.^12^ Identifying patterns of atrophy that help to distinguish pathological processes from each other (and from normal ageing), based on gold standard histopathology, may provide greater understanding of the ability of clinical imaging to predict dementia pathology and help to translate imaging features, identified through research, into clinically useful biomarkers.

To date, relatively few imaging studies using voxelwise approaches are based on cases with histopathological confirmation of diagnosis, and those who have typically used small sample sizes. Furthermore, many studies have made comparisons only between people with dementia and healthy controls (see table 1 for a review of the literature), whereas in clinical practice, structural imaging is increasingly used to distinguish between the different pathological forms of dementia. Motivated by this clinical question, we performed a comprehensive computational analysis of brain volume loss in the largest series yet reported, including 186 individuals with a diagnosis of dementia during life and primary histopathological diagnosis of one of six pathologies (Alzheimer’s disease (AD), dementia with Lewy bodies (DLB), 3-repeat-tau, 4-repeat-tau, TDP43A and TDP43C (transactive response DNA-binding protein 43)). The aim of this study was to identify patterns of atrophy that would not only differentiate these primary neurodegenerative pathology groups from cognitively healthy control subjects but, critically, also from one another.

**Table 1:** Voxel-based morphometry studies in pathology-confirmed (or genetically confirmed) dementias

Study	Year	Defined pathologies	Pathology confirmed cases, n	Comparison with controls	Direct pathology comparison	PubMed ID
Whitwell *et al*	2004	Tau-positive FTLD, tau-negative FTLD	9, 8	Yes	No	16908994
Whitwell *et al*	2005	FTLD-U, Pick’s, MAPT	9, 7, 5	Yes	Yes	16157747
Josephs *et al*	2006	NA—various pathologies grouped by language impairment features	12	NA	NA	16613895
Grossman *et al*	2007	Tau-positive FTLD, tau-negative FTLD, (frontal variant)-AD	5, 4, 3	Yes	No	17998442
Josephs *et al*	2008a	(aphasic)-AD, (aphasic)-FTLD-U, (typical)-AD	5, 5, 10	Yes	Yes	18166704
Josephs *et al*	2008b	PSP, CBD	13, 11	Yes	No	17097770
Josephs *et al*	2008c	Argyrophilic grain pathology	12	Yes	No	17188783
Whitwell *et al*	2008	Braak stages III–VI	10, 13, 32, 27	Yes	NA	18765650
Josephs *et al*	2009	NA—FTLD groups defined based on clinical features	11	NA	NA	19884571
Pereira *et al*	2009	Tau-positive FTLD, FTLD-U	6, 9	Yes	No	19433738
Hu *et al*	2010	NA—various pathologies grouped by language impairment features	8	NA	NA	20713948
Josephs *et al*	2010	(CBS)-CBD, (CBS)-AD	6, 5	Yes	Yes	20629131
Rohrer *et al*	2010a	GRN, MAPT	9, 11	Yes	Yes	20045477
Rohrer *et al*	2010b	FTLD-TDP43 (types A–C and unspecified)	9, 5, 10, 4	Yes	No	21172843
Whitwell *et al*	2010a	(CBS)-TDP43, (CBS)-AD, (CBS)-CBD, (CBS)-PSP	5, 6, 7, 6	Yes	Yes	21098403
Whitwell *et al*	2010b	FTLD-TDP43 (types A–C)	22, 9, 11	Yes	Yes	21172844
Lee *et al*	2011	CBD, (CBS)-AD, (CBS)-CBD, (CBS)-PSP, (CBS)-TDP, (CBS)-mixed	18, 9, 14, 5, 5, 5	Yes	Yes	21823158
Rankin *et al*	2011	(bvFTD)-CBD, (bvFTD)-Pick’s	5, 5	Yes	Yes	21881831
Rohrer *et al*	2011	Pick’s, MAPT, CBD, TDP43A, TDP43C	9, 6, 5, 6, 12	Yes	No	21908872
Whitwell *et al*	2011a	(typical)-AD, (atypical)-AD, FTLD	14, 14, 14	Yes	Yes	19914744
Whitwell *et al*	2011b	Pick’s, CBD, TDP43A	5, 5, 5	Yes	Yes	21556732
Boxer *et al*	2012	NA—FTLD and AD correlations with saccade parameters	37	NA	NA	22491196
Hornberger *et al*	2012	(bvFTD)-FTLD, AD	19, 18	Yes	No	23012333
Khan *et al*	2012	(slowly progressive bvFTD)-C9ORF72	2	Yes	No	22399793
Mahoney *et al*	2012	C9ORF72, MAPT, GRN	11, 11, 8	Yes	Yes	22366791
Whitwell *et al*	2012a	NA—bvFTD groups defined based on frontal lobe symmetry	80	NA	NA	22502999
Whitwell *et al*	2012b	MAPT, GRN, C9ORF72, FTLD	25, 12, 19, 12	Yes	Yes	22366795
Whitwell *et al*	2012c	typical AD, hippocampal-sparing AD, limbic-predominant AD	125, 19, 33	Yes	Yes	22951070
Cash, Ridgway *et al*	2013	(presymptomatic)-familial AD, (symptomatic)-familial AD	69, 50	Yes	Yes	24049139
Caso *et al*	2013	Pick’s	1	Yes	No	22713404
Coon *et al*	2013	FTLD-MND	2	Yes	No	22051030
Scahill *et al*	2013	APP, PSEN1	10, 18	Yes	Yes	23380992
Toledo *et al*	2013	AD, AD+MTL, AD+DLB, AD+DLB+MTL	7, 5, 6, 4	No	Yes	24252435
Josephs *et al*	2013a	(typical)-AD, (lvPPA)-AD	20, 10	Yes	No	23541297
Josephs *et al*	2013b	CSTD-positive TDPC, CSTD-equivocal TDPC, CSTD-negative TDPC	2, 5, 5	Yes	Yes	23358603
Murray *et al*	2013	NA—DLB groups defined by presence of REM sleep behaviour disorder	75	NA	NA	24107861
Caso *et al*	2014	(nfvPPA)-tau, (nfvPPA)-TDP43	9, 2	Yes	No	24353332
Henry *et al*	2014	TDP43C	1	Yes	No	23171151
Josephs	2014	TDP43-positive AD, TDP43-negative AD	195, 147	Yes	Yes	24659241
Nedelska *et al*	2015	DLB, DLB-AD, AD	20, 22, 30	Yes	Yes	25128280
Ossenkoppele *et al*	2015	(behavioural/dysexecutive)-AD, (typical)-AD, (bvFTD)-FTLD	24, 17, 12, 8, 21	Yes	Yes	26141491
Sala-Llonch *et al*	2015	Symptomatic-PSEN1, asymptomatic-PSEN1	11, 13	Yes	No	25638532
Shingawa *et al*	2015	NA—C9ORF72 groups were defined based on the presence of delusions	17	NA	NA	25342578

Parentheses indicate additional clinical features or diagnosis. The number of pathology cases is listed per defined pathology group.

AD, Alzheimer’s disease; APP, amyloid precursor protein mutation carriers; bvFTD, behavioural variant FTD; C9ORF72, chromosome 9 open reading frame 72 mutation carriers; CBD, corticobasal degeneration; CBS, corticobasal syndrome; CTSD, corticospinal tract degeneration; DLB, dementia with Lewy bodies; FTD, frontotemporal dementia; FTLD, frontotemporal lobar degeneration; FTLD-MND, FTLD with motor neuron disease; FTLD-U, FTLD with ubiquitin-positive (tau-synuclein-negative and alpha-synuclein-negative) inclusions; GRN, progranulin mutation carriers; MAPT, microtubule-associated protein tau mutation carriers; MTL, medial temporal lobe pathology; NA, not applicable; nfvPPA, non-fluent variant primary progressive aphasia; PSEN1, presenilin-1 mutation carriers; PSP, progressive supranuclear palsy; TDP43, transactive response DNA-binding protein 43.

## Materials and methods

### Study population

Two hundred and fifteen people were identified with a usable T1-weighted MRI, a diagnosis of dementia during life and post-mortem (n=206) or biopsy (n=9) confirmation of the underlying pathology. Twenty-one individuals were excluded for having incomplete or inconclusive pathology, and eight individuals were excluded due to insufficient data to study them as a group (n=4 amyloid precursor protein mutation carriers, n=1 TDP43B, n=3 with fused in sarcoma pathology). A total of 186 individuals were included in the subsequent analysis: 107 had a primary pathology diagnosis of AD (68 early-onset (<65 years at disease onset), 29 late-onset (**≥**65 years at disease onset), 10 presenilin-1 mutation carriers), 25 with DLB, 11 with 3-repeat-tau, 17 with 4-repeat-tau, 12 TDP43A, and 14 TDP43C. Pathological examination of brain tissue was carried out between 1997 and 2015 according to standard histopathological processes and criteria in use at the time of assessment at one of four centres: the Queen Square Brain Bank, London; Kings College Hospital, London; VU Medical Centre, Amsterdam and Institute for Ageing and Health, Newcastle. The study included 73 cognitively normal control subjects (based on clinical diagnosis) who were recruited to various imaging studies that had been carried out at the Dementia Research Centre, London. Controls were separated into younger (<65 years of age at the time of scan, n=33) and older (**≥**65 years of age at the time of scan, n=40) groups to better match the patient groups. All patients or their legal representatives consented, during life, to brain donation and use of their clinical data for research purposes at each of the participating sites. Ethical approval for this retrospective study was obtained from the National Research Ethics Service Committee London-South East. Group demographics were compared using Kruskal-Wallis tests, with a significant result being followed by post hoc pairwise multiple comparisons testing using Dunn’s test. A subset of these subjects was included in earlier work focusing on visual rating scales.^13^ Data processing and analyses were performed using Python libraries NumPy 1.8.1 (http://www.numpy.org/), SciPy 0.14.0 (https://www.scipy.org/scipylib/index.html) and Pandas 0.14.1 (http://pandas.pydata.org/) on Python 2.7.6–64-bit.

### MRI scanning

All individuals had T1-weighted volumetric MRI performed during life. As the data were collected retrospectively from multiple centres, the images were acquired on scanners from three different manufacturers (Philips, GE, Siemens) using a variety of different imaging protocols. Magnetic field strength varied between 1.0 T (n=15 scans), 1.5 T (n=201 scans) and 3 T (n=43 scans).

### Image analysis

Voxel-based morphometry (VBM) preprocessing and analysis was performed using SPM12b (Statistical Parametric Mapping, V.12b revision 5829; http://www.fil.ion.ucl.ac.uk/spm) and Matlab V.R2012a (7.14.0.739–64-bit, uk.mathworks.com/products/matlab/). Due to the variability in scanning parameters, an initial rigid registration to the Montreal Neurological Institute International Consortium for Brain Mapping 152 (ICBM152) template was performed using the Reg-Aladin tool from the NiftyReg package (https://sourceforge.net/projects/niftyreg/) to provide a better starting point for the SPM preprocessing pipeline. Each registration was then checked and manually adjusted (if necessary) such that the anterior commissure was within a few millimetres of the origin and the orientation was within a few degrees of the ICBM152 template. Grey matter, white matter and cerebrospinal fluid (CSF) segmentations were obtained using the unified segmentation approach^14^ with default settings. A group average tissue probability map was generated through iterative alignment of the initial grey and white matter segmentations to an evolving estimate of their groupwise average using the Dartel toolbox.^15^ The initial grey and white matter segmentations were then warped using the Dartel transformations and modulated to account for local volume changes, then smoothed with a 6 mm full width at half maximum Gaussian kernel. Grey and white matter masks were created based on the optimal threshold of the group average segmentations using the automatic mask creation strategy in the SPM Masking toolbox (http://www0.cs.ucl.ac.uk/staff/g.ridgway/masking/).

The imaging data were assessed by applying the general linear model at the level of each voxel using all images (n=259) and modelling the following terms: a 10-level group factor (younger controls, older controls, early-onset AD, presenilin-1 mutation carriers, late-onset AD, DLB, 3-repeat-tau, 4-repeat-tau, TDP43A and TDP43C); factors representing magnetic field strength, imaging site and sex; and covariates for age at the time of scanning and total intracranial volume (computed by summing up probabilistic voxel volumes in grey matter, white matter and CSF segmentations). The model was used to investigate the pairwise contrasts between the primary pathology groups, with the AD pathology group stratified by age at disease onset and genetic mutation status. The groups were also contrasted with age-matched controls (ie, older controls were contrasted with the late-onset AD and DLB groups; younger controls were contrasted with all other groups). Correction for multiple comparisons was made using random field theory (for peak height) to control the familywise error (FWE) rate at a significance level of p<0.001, or 0.05 in most cases, although some results are shown at an uncorrected level of p<0.001. Unthresholded effect size maps are also displayed to allow better characterisation of brain volume loss that did not reach statistical significance.

## Results

### Study population

Demographic details of the patients and control subjects are presented in table 2. As expected, the late-onset AD group and the DLB group were, on average, older than the other groups at disease onset, and therefore, at the time of scanning, although this did not reach statistical significance in all cases (see online supplementary table 1). Between-group testing of time from scan until death was statistically significant (p<0.05) with the DLB group having the shortest mean interval (3.7 years) and the TDP43C group with the longest mean interval (7.0 years); however, these differences did not survive multiple comparisons correction. There were no statistically significant differences in sex (p=0.05), disease duration (p=0.13), Mini-Mental State Examination within 12 months of scanning (p=0.24) or total intracranial volume (p=0.20) between the pathology groups.

10.1136/jnnp-2016-314978.supp1 supplementary table 1

**Table 2:** Patient demographics

	Younger controls	Older controls	EOAD	PSEN1	LOAD	DLB	3R-tau	4R-tau	TDP43A	TDP43C
N	33	40	68	10	29	25	11	17	12	14
Sex (%male)	30	70	65	60	66	72	72	53	50	50
Age at onset (years)	NA	NA	55.3 (5.7)	39.6 (4.6)	71.7 (6.0)	67.1 (6.1)	57.2 (6.3)	61.4 (9.7)	58.2 (6.5)	61.6 (8.0)
Age at scan (years)	59.9 (4.8)	72.2 (5.2)	60.5 (6.2)	44.0 (4.2)	75.8 (5.7)	71.3 (6.0)	62.6 (6.2)	65.7 (9.3)	60.3 (7.7)	66.0 (6.6)
Disease duration at scan (years)	NA	NA	5.2 (2.9)	4.4 (2.3)	4.1 (2.1)	4.2 (2.9)	5.4 (2.1)	4.4 (2.4)	2.9 (2.4)	4.5 (3.0)
MMSE (×/30)	NA	NA	18 (7)	15 (6)	19 (5)	20 (5)	21 (7)	23 (5)	17 (8)	23 (6)
Time from scan until death (years)	NA	NA	5.8 (2.9)	4.2 (2.2)	5.7 (3.3)	3.7 (2.3)	6.0 (3.5)	4.8 (2.3)	5.2 (4.0)	7.0 (3.4)
TIV (mL)	1425 (121)	1531 (156)	1504 (155)	1440 (163)	1484 (136)	1530 (149)	1506 (164)	1517 (178)	1480 (133)	1483 (165)
1.5 T scans	79%	73%	77%	80%	86%	76%	91%	76%	50%	86%
3.0 T scans	21%	27%	13%	20%	7%	8%	9%	24%	33%	7%

Data are reported as mean (SD).

DLB, dementia with Lewy bodies; EOAD, early-onset Alzheimer’s disease; LOAD, late-onset Alzheimer’s disease; MMSE, Mini-Mental State Examination (within 12 months of scanning); NA, not applicable; PSEN1, presenilin-1 mutation carriers; TDP43, transactive response DNA-binding protein 43; TIV, total intracranial volume.

### Comparison with age-matched controls

**Figure 1:**
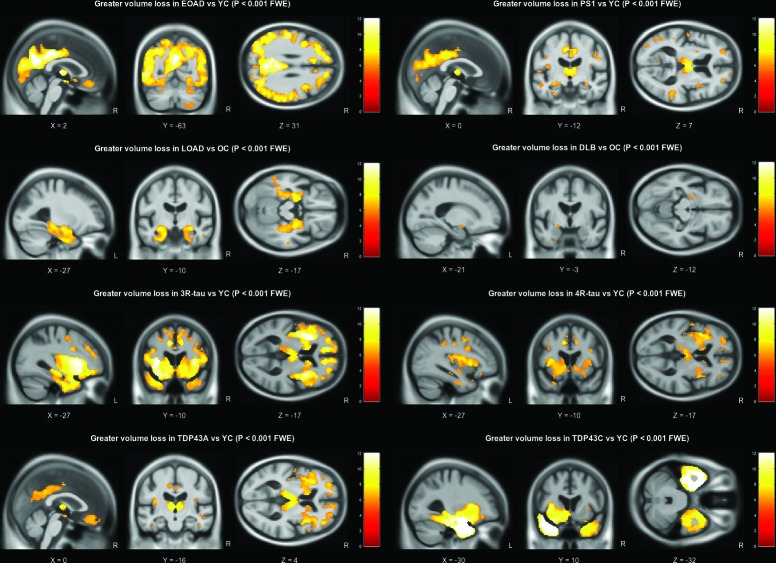
Grey matter volume differences based on pairwise comparison with the appropriate age-matched control group. All results are shown at a familywise error corrected significance level of p‌<0.001. Brain slices are displayed based on peak voxel location. DLB, dementia with Lewy bodies; EOAD, early-onset Alzheimer’s disease; FTLD, frontotemporal lobar degeneration; L, left; LOAD, late-onset Alzheimer’s disease; OC, older controls; R, right; YC, younger controls.

**Figure 2:**
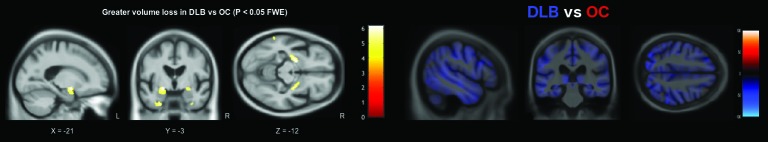
DLB versus older controls. Grey matter volume differences based on pairwise comparison between the DLB and older control groups. The statistical parametric map is shown on the left with brain slices displayed based on peak voxel location. Results are thresholded at a significance level of p<0.05 familywise error corrected. The axial effect size map is shown on the right, regions in blue represent greater volume loss in the DLB group. DLB, dementia with Lewy bodies; FWE, familywise error rate; L, left; OC, older controls; R, right.

Using a strict statistical threshold (p<0.001 FWE), significant differences in grey matter volume were found between each of the pathology groups and the appropriate age-matched control group (figure 1). The early-onset AD group demonstrated a diffuse pattern of atrophy, predominantly affecting the parietal and temporal cortex, with some additional extension into the frontal lobes. The presenilin-1 subgroup had similar parietal extension with more localised volume loss in the thalamus, medial temporal lobe and temporal neocortex. Compared with older control subjects, the late-onset AD group had a more focal pattern of grey matter volume loss in the medial temporal lobes, particularly the hippocampi. The DLB group also demonstrated significant medial temporal lobe atrophy when compared with older controls; however, this was much less extensive than the changes seen in the AD group, affecting the amygdalae (bilaterally, although worse on the left), the region of the left choroid fissure and a very small region around the lateral superior temporal gyrus. Relaxing the statistical threshold to p <0.05 FWE (figure 2), further volume differences were seen in the region of the right amygdala and choroid fissure; however, extension into other temporal lobe regions was confined to the left hemisphere. The frontotemporal lobar degeneration (FTLD)-tau groups had extensive grey matter volume loss in the frontal lobe affecting the superior, middle and inferior frontal gyri, extending into the insula. However, the right hemisphere was less severely affected in the 4-repeat-tau group, whereas the 3-repeat-tau group showed greater extension into the anterior temporal lobes. The TDP43A group demonstrated a similar, more symmetrical pattern of frontal volume loss, with additional volume loss extending into parietal lobe regions. The thalamic region was significantly affected in all three FTLD pathologies (3-repeat-tau, 4-repeat-tau and TDP43A). The TDP43C group had a significant volume loss in the temporal lobe, particularly anteriorly and in the left hemisphere where it extended into the frontal piriform and insular cortex. The reverse contrast was also investigated in all groups but showed no statistically significant regions where volume loss was more pronounced in the control group.

### Comparison between pathology groups

**Figure 3:**
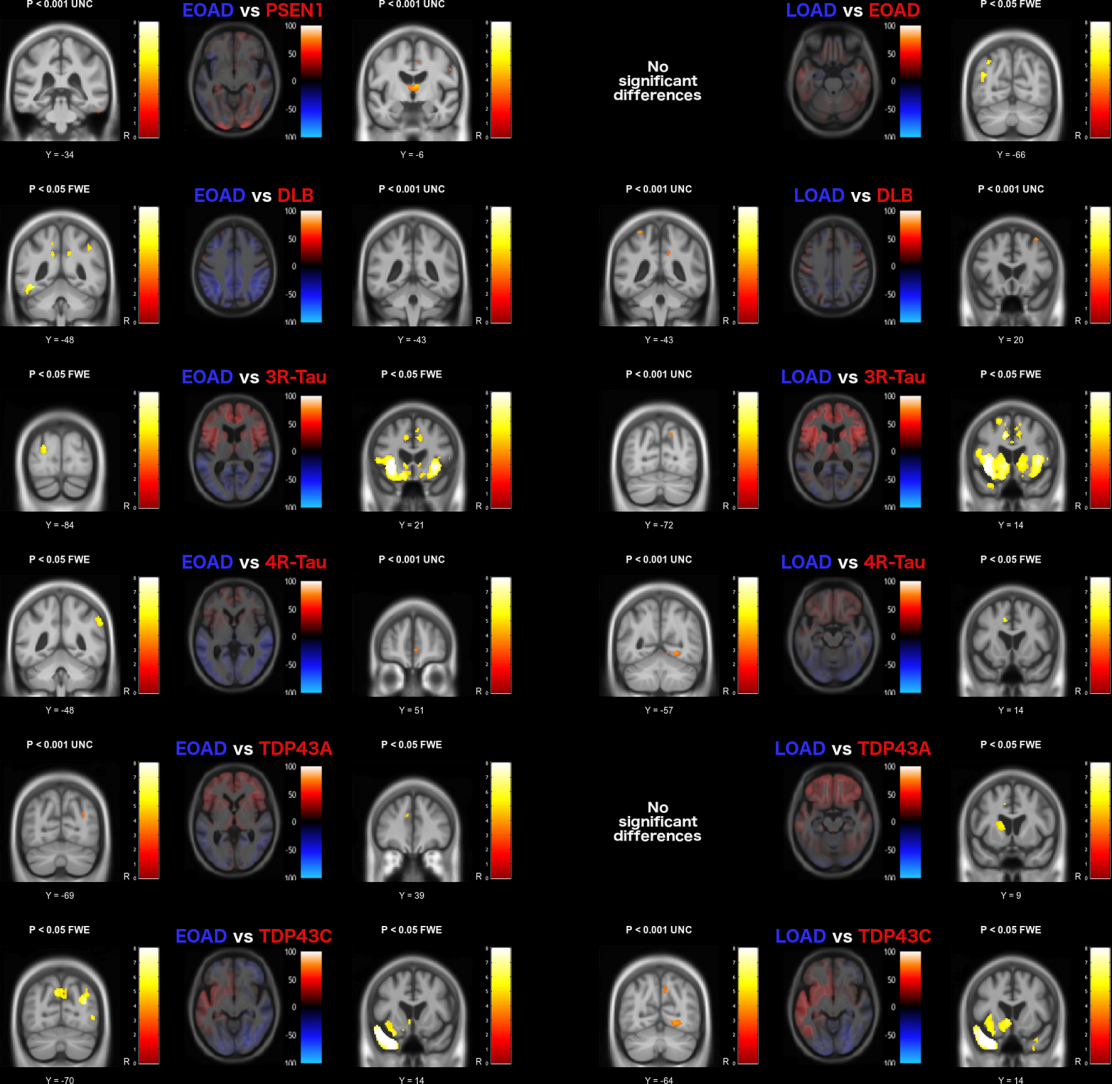
Grey matter volume differences based on pairwise comparison with the Alzheimer’s disease pathology groups. The axial image slices are effect size maps and the label shown above each map indicates the group comparison. Text colour relates to image colour, with the coloured regions in the image representing greater volume loss in the associated group, and brain slices chosen to represent the most interesting findings. The statistical parametric map slices to the left and right of the effect size maps represent the forward and reverse contrasts associated with each group comparison, with brain slices displayed based on peak voxel location. Greater volume loss in the blue group is shown on the left, with the red group shown on the right statistical parametric map. DLB, dementia with Lewy bodies; EOAD, early-onset Alzheimer disease; FTLD, frontotemporal lobar degeneration; FWE, familywise error rate; L, left; LOAD, late-onset Alzheimer’s disease; R, right; UNC, uncorrected (for multiple comparisons).

**Figure 4:**
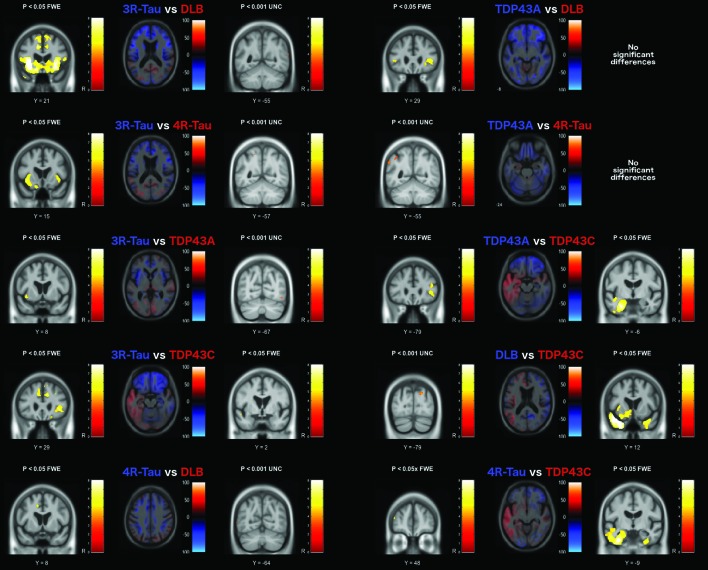
Grey matter volume differences based on pairwise comparison with the FTLD pathology groups. The axial image slices are effect size maps and the label shown above each map indicates the group comparison. Text colour relates to image colour, with the coloured regions in the image representing greater volume loss in the associated group, and brain slices chosen to represent the most interesting findings. The statistical parametric map slices to the left and right of the effect size maps represent the forward and reverse contrasts associated with each group comparison, with brain slices displayed based on peak voxel location. Greater volume loss in the blue group is shown on the left, with the red group shown on the right statistical parametric map. DLB, dementia with Lewy bodies; FTLD, frontotemporal lobar degeneration; FWE, familywise error rate; L, left; R, right; UNC, uncorrected (for multiple comparisons).

Differences in grey matter volume based on pairwise comparisons of each of the pathology groups are presented in figures 3 and 4. The early-onset AD group had significant grey matter volume loss in parietal lobe regions (p<0.05 FWE) compared with the other groups (figure 2); however, in comparison with the presenilin-1 and TDP43A groups, the differences were very small and did not survive correction for multiple comparisons.

The presenilin-1 group had greater volume loss in the thalamus bilaterally compared with the early-onset AD group (p<0.001 uncorrected), and around the cingulate gyrus in comparison with both the early-onset and late-onset AD group (p<0.05 FWE) and the DLB group (p<0.05 FWE). In comparison with the FTLD groups, greater volume loss was observed in more posterior brain regions, also around the cingulate gyrus when contrasted with the 3-repeat-tau (p<0.05 FWE) and TDP43C groups (p<0.05 FWE), and around the superior parietal lobule when contrasted with the 4-repeat-tau (p<0.05 FWE) and TDP43A groups (p<0.001 uncorrected).

The late-onset AD group also demonstrated significant volume loss in parietal and occipital cortical regions in comparison with DLB, tau and TDP43C groups (p<0.001 uncorrected). In addition, the associated effect size maps also highlighted smaller hippocampal volumes in all but the 3-repeat-tau comparison and the left hippocampus when compared with the TDP43C pathology group.

On the basis of the effect size maps, the DLB group demonstrated greater volume loss in the region of the precentral and postcentral gyri, the precuneus and the cerebellum in comparison with all other groups; however, none of these differences remained after correction for multiple comparisons.

The FTLD pathology groups demonstrated greater and extensive volume loss in frontal and temporal lobe regions in comparison with all other groups (figures 3 and 4). The 3-repeat tau group had significantly smaller grey matter volume in the frontal cortex in comparison with all other pathology groups (p<0.05 FWE, figures 3 and 4), particularly around the piriform and insular cortex, and around the anterior cingulate gyrus except in comparison with the 4-repeat-tau and TDP43A groups. The 4-repeat-tau group also had significant volume loss around the anterior cingulate, although this did not survive correction for multiple comparisons for all contrasts. The TDP43A group demonstrated greater volume loss around the lateral orbito-frontal and inferior-frontal gyrus in comparison with the DLB and TDP43C groups, while in comparison with the late-onset AD group, volume differences were localised to the left anterior striatum (p<0.05 FWE). As expected, the TDP43C group showed extensive grey matter volume loss in the left anterior temporal lobe in comparison with all other pathology groups, extending into the right anterior temporal lobe in comparison with the late-onset AD group, the DLB and the 4-repeat-tau groups (p<0.05 FWE).

**Figure 5:**
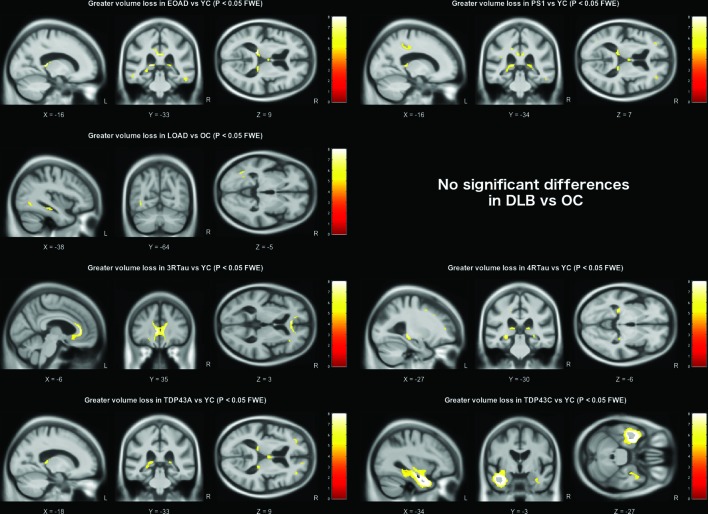
White matter volume differences based on pairwise comparison with the appropriate age-matched control group. All results are shown at a familywise error corrected significance level of p<0.05. Brain slices are displayed based on peak voxel location. DLB, dementia with Lewy bodies; EOAD, early-onset Alzheimer’s disease; FTLD, frontotemporal lobar degeneration; FWE, familywise error rate; L, left; LOAD, late-onset Alzheimer’s disease; OC, older controls; PS1, presenilin-1; R, right; YC, younger controls.

While it is only possible to show a few representative brain slices in each figure (figures 3–5), unthresholded statistical maps have been uploaded to the NeuroVault repository (http://neurovault.org/collections/ADHMHOPN/) to allow for further inspection of grey matter and white matter volume differences throughout the brain for each comparison.

## Discussion

To our knowledge, this is the largest VBM imaging study to investigate brain volume differences between different pathologically proven dementias, allowing for a comprehensive assessment of differences between pathology groups and healthy controls, as well as direct comparisons between pathology groups (see table 1 for a review of the existing literature). Care was taken to age-match the patient groups to controls in order to reduce the influence of age-related volume loss, and results were also statistically adjusted for age (at time of scan), sex and total intracranial volume,^16^ as well as MRI field strength and site. Individuals with AD pathology were also separated into autosomal-dominant presenilin-1 mutation carriers, early-onset (before 65 years) and late-onset groups to investigate patterns of atrophy reported in relation to these often quite distinct disease phenotypes.^17 18^ Individuals with FTLD-tau were stratified based on the predominant tau isoform found at post-mortem. In comparison with healthy controls, results are reported based on a strict statistical threshold, controlling the FWE rate at a significance level of p<0.001 (ie, fewer than 1 in 1000 statistical maps would have any false-positive voxels).

AD pathology was associated with extensive volume loss, particularly in temporoparietal regions (figure 1), in keeping with the known locus of AD pathology. However, the early-onset group demonstrated more extensive parietal lobe atrophy, whereas the late-onset group demonstrated more focal medial temporal lobe volume loss (figure 1). In the direct comparison between the two groups, only volume differences in the left parietal lobe were statistically significant. This confirms previous reports from smaller studies based on clinical diagnosis.^19^ This, in turn, has clinical implications, confirming that, despite research criteria that focus on the presence/absence of hippocampal atrophy for the diagnosis of AD, this may not be a strong feature in patients with early-onset disease.

The late-onset AD group also demonstrated smaller parietal/occipital lobe volumes in comparison with the DLB, FTLD-tau and TDP43C groups (figures 2 and 3), in keeping with reported disruption to the brain’s default mode network due to AD pathology.^20^ This was also evident in the other Alzheimer’s pathology groups, with additional involvement of the anterior cingulate cortex.

In comparison with healthy controls and the early-onset AD group, the autosomal-dominant mutation carriers demonstrated focal volume loss in thalamic regions, which has recently been described as a potentially critical hub to explain the symptomology of the disease.^21^ Increased volume loss in this region has previously been reported in presymptomatic and symptomatic presenilin-1 mutation carriers compared with controls^18 22^ but not between presenilin-1 and sporadic early-onset AD as shown here.

Similar to the other neurodegenerative pathologies included in this study, patients with primary DLB pathology demonstrated global volume loss in comparison with the age-matched controls (see effect size map in figure 2). However, when contrasted directly with the other groups, the effect was much smaller, with relative sparing of the hippocampi, consistent with previous reports.^23–26^ Statistical significance was reached in the region of the amygdalae bilaterally. However, volume reduction in this region also correlates with neurofibrillary tangle burden, which may explain why significant differences were not seen in the direct comparison of DLB with AD.^23 27^ When compared with the other pathologies, DLB was associated with reduced grey matter volume in the region of the precentral and postcentral gyri. Similar findings have been reported in Parkinson’s disease, suggesting that atrophy in this region may be a feature of Lewy body pathology more generally.^28^ The DLB group also demonstrated occipital lobe volume loss, in keeping with previous reports of occipital hypometabolism revealed using fluorodeoxyglucose-positron emission tomography.^23 29^ While primary motor, sensory and occipital cortex changes are often considered relatively spared in most degenerative dementias, these results suggest that there may be subtle but detectable changes in these regions in DLB; this, in turn, provides clear hypotheses that can be tested using more advanced MRI techniques.

There was considerable overlap in the pattern of frontal lobe atrophy between the FTLD-tau groups and the TDP43A group,^30 31^ particularly in the ventrolateral prefrontal cortex. However, the TDP43A pathology group appeared to have a more symmetrical presentation with greater parietal extension, while the left hemisphere was more affected in the 3-repeat-tau and 4-repeat-tau groups. The TDP43C group, which predominantly manifests clinically as semantic dementia, had extensive left anterior temporal lobe atrophy as previously reported in other studies.^32 33^

Although this study benefits from an unprecedented sample size of ante-mortem imaging acquired in patients with pathological confirmation of their diagnosis, analysis using the most advanced SPM software and, where possible, strict statistical thresholding, it has a number of limitations. Including data from multiple sites increases the sample size, the power to detect statistical differences between groups and importantly the generalizability of the findings, but it also introduces potential confounds relating to the different MRI scanners and pulse sequences used to acquire the images. To account for this, magnetic field strength, which is likely to be the most significant source of variability,^34^ was included along with acquisition site as factors in the model. Although the overall sample size is larger than those previously reported in the literature, sample sizes were insufficient to stratify beyond the primary pathological diagnosis, noting that in practice many patients will have multiple neurodegenerative pathologies of varying extent and severity, and concomitant vascular disease, all of which may influence developing patterns of brain volume loss.^35 36^ In addition, control subjects were not pathologically confirmed; therefore, we cannot rule out presymptomatic pathology in this group, which may reduce the power in comparing the pathology groups with controls. However, this does not affect the more clinically relevant between-pathology group comparisons. Although not quite reaching statistical significance (p=0.05), there was a lower proportion of men included in the younger control group than the other groups; however, as all subjects were included in a single model, sex differences are adjusted for using precise estimates from the total number of men and women included in the model. Finally, as with many research studies, there may be selection bias in the patients included in this cohort: those patients with memory and language led presentations, and those with familial forms of dementia, are more likely to take part in research, including brain donation, than patients with more challenging behavioural problems, for example. This may affect the representation of pathologies in this cohort and reduce how well the results generalise to the wider population.

Although the advent of molecular imaging provides a direct means of identifying underlying pathology during life,^37 38^ the ability to use information from widely available, routinely acquired, noninvasive MRI scans is currently much more applicable in clinical practice; and structural MRI continues to be the most widely used surrogate marker of neurodegeneration, reflected by its inclusion in a recently proposed classification scheme,^39^ and its continued inclusion in research diagnostic guidelines.^40^ Ultimately, however, it is only by using robust methodologies to compare in vivo tests with post-mortem confirmation in large and varied data sets that the diagnostic utility of any dementia biomarker can be assessed. The data from this study—which have been uploaded to the NeuroVault repository for independent scrutiny—provide much needed validation of the regions of the brain where neurodegenerative pathologies are detectable during life, and as such may have greater utility in clinical decision making. In addition, this study highlights where evidence is lacking and demonstrates that voxelwise brain mapping techniques applied to well-defined groups can reveal potentially overlooked regions that warrant further investigation as imaging biomarkers.
