# Supplementary material for: Patterns of atrophy in pathologically confirmed dementias: a voxelwise analysis
Source: J Neurol Neurosurg Psychiatry. 2017 May 4;88(11):908–16. doi: 10.1136/jnnp-2016-314978 (PMC5740544; doi:10.1136/jnnp-2016-314978)
Supplement: supplementary table 1 [file jnnp-2016-314978supp001.pdf]

	Younger Controls	Older Controls	EOAD	PSEN1	LOAD	DLB	3RTau	4RTau	TDP43A	TDP43C
										**Age at Onset**
**Younger Controls**		NA	X	X	NA	NA	X	X	X	X
**Older Controls**	NA		NA	NA	X	X	NA	NA	NA	NA
**EOAD**	X	NA		X	$P<0.05$	$P<0.05$	X	X	X	X
**PSEN1**	X	NA	X		$P<0.05$	$P<0.05$	X	$P<0.05$	X	X
**LOAD**	NA	X	$P<0.05$	$P<0.05$		X	$P<0.05$	X	X	X
**DLB**	NA	X	$P<0.05$	$P<0.05$	X		X	X	X	X
**3RTau**	X	NA	X	X	$P<0.05$	X		X	X	X
**4RTau**	X	NA	X	$P<0.05$	$P<0.05$	X	X		X	X
**TDP43A**	X	NA	X	X	$P<0.05$	$P<0.05$	X	X		X
**TDP43C**	X	NA	X	$P<0.05$	$P<0.05$	X	X	X	X	
	**Age at Scan**									
